# Retraction: Regulation of circGOLPH3 and its binding protein CBX7 on the proliferation and apoptosis of prostate cancer cells

**DOI:** 10.1042/BSR-2020-0936_RET

**Published:** 2024-05-17

**Keywords:** apoptosis, CBX7, cell proliferation, circGOLPH3, prostate cancer

This article is being retracted from *Bioscience Reports* at the request of the Editor-in-Chief and the Editorial Board following receipt of a notification from a reader, alerting the Editorial Board to Figure 2B in this paper which appears as Figure 1C in a previous publication by Jiao et al, 2018 (DOI: 10.3390/ijms19010271) with panel rearrangements. The Editorial Office also have additional concerns regarding the figures in this paper, specifically:

- Regions of Figure 3A OE-circGOLPH3 panel which appear similar to regions of Figure 3A OE-NC panel, and separately regions of Figure 3A si-circGOLPH3 panel which appear similar to regions of Figure 6A si-NC panel
- Figure 2A si-NC panel which seems to appear as Figure 5A OE-CBX7 panel

The authors have been contacted with regards to the retraction and have not responded to the Journal's queries or the concerns raised. Given the extent of the issues raised, the Editorial Board stand by the decision to retract the article.

